# Value of gadoxetic acid-enhanced MR imaging for preoperative prediction of future liver regeneration after hemihepatectomy

**DOI:** 10.1007/s11604-024-01629-w

**Published:** 2024-08-16

**Authors:** Nobuhiro Fujita, Yasuhiro Ushijima, Masahiro Itoyama, Daisuke Okamoto, Keisuke Ishimatsu, Kosuke Tabata, Shinji Itoh, Kousei Ishigami

**Affiliations:** 1 Department of Clinical Radiology, Graduate School of Medical Sciences, Kyushu University, 3-1-1 Maidashi, Higashi-ku, Fukuoka, 812-8582 Japan; 2 Departments of Surgery and Science, Graduate School of Medical Sciences, Kyushu University, 3-1-1 Maidashi, Higashi-ku, Fukuoka, 812-8582 Japan

**Keywords:** Gadoxetic acid, MRI, Liver regeneration, Hepatectomy

## Abstract

**Purpose:**

Liver resection is currently considered the most effective treatment for patients with liver cancer. To the best of our knowledge, no study has investigated the association between gadoxetic acid-enhanced magnetic resonance imaging (MRI) findings and liver regeneration in patients who underwent hemihepatectomy. We aimed to clarify the relationship between the signal intensity (SI) of the liver parenchyma on gadoxetic acid-enhanced MRI and the degree of liver regeneration in patients who underwent hemihepatectomy.

**Materials and methods:**

Forty-one patients who underwent gadoxetic acid-enhanced MRI before hemihepatectomy were enrolled. We calculated the liver-to-erector spinae muscle SI ratio (LMR) in the hepatobiliary phase and the precontrast images. ΔLMR was calculated using the following equation: ΔLMR = (LMR in the hepatobiliary phase−LMR in the precontrast image)/LMR in the precontrast image. The preoperative and postoperative remnant liver volumes (LVs) were calculated using CT volumetry. We calculated the resection rate (RR) and liver regeneration index (LRI) using the following formulas: RR = Resected LV/Total LV × 100 and LRI = (postoperative remnant LV−preoperative remnant LV)/preoperative remnant LV × 100. The relationships among LRI, imaging, and clinicopathological factors were analyzed.

**Results:**

Univariate analysis showed RR and ΔLMR showed a positive correlation with LRI (ρ = 0.4133, p = 0.0072 and ρ = 0.7773, p < 0.001, respectively). Spleen volume showed a negative correlation with LRI (ρ = −0.3138, p = 0.0486). Stepwise multiple regression analysis showed ΔLMR and RR were independently correlated with LRI (β coefficient = 44.8771, p = 0.0198 and β coefficient = 1.9653, p < 0.001, respectively).

**Conclusion:**

ΔLMR may serve as a preoperative predictor of liver regeneration in patients undergoing hemihepatectomy.

## Introduction

Liver cancer is one of the most common causes of death worldwide, particularly in East Asia [1]. Liver resection is currently considered the most effective treatment for patients with liver cancer [2]. Although the liver can be regenerated by hyperplasia after hepatectomy, liver regeneration (LR) can be inhibited if impairment or injury exceeds the liver’s ability for regeneration [3]. For this reason, despite the recent technological developments in liver surgery, liver failure is one of the major complications of major hepatectomy, with a reported incidence of 0.7 to 9.1%, which may be related to inadequate LR [4, 5]. Therefore, estimating postoperative LR is particularly important in patients undergoing major hepatectomies to ensure safety and improve clinical outcomes [6].

Poor LR is closely associated with underlying liver diseases, including liver dysfunction and fibrosis [7–9]. Gadoxetic acid is a hepatobiliary-specific contrast agent that is taken up by functioning hepatocytes in the hepatobiliary phase [10, 11]. The uptake of gadoxetic acid in the liver parenchyma can directly reflect the function of the liver, and it has been reported that the uptake of gadoxetic acid into the liver parenchyma decreases with liver dysfunction [12, 13] and liver fibrosis progression [14]. Thus, we surmised that the uptake of gadoxetic acid in the liver parenchyma could be used as an indicator of LR. However, to the best of our knowledge, no study has investigated the association between gadoxetic acid-enhanced MRI findings and LR in patients who underwent hepatectomy.

Accordingly, this study aimed to clarify the relationship between MRI parameters and the degree of LR who underwent hemihepatectomy, with a special focus on gadoxetic acid uptake in the liver parenchyma. Sub-analyses were also conducted based on clinical and pathological liver functions.

## Materials and methods

### Patients

**Fig. 1:**
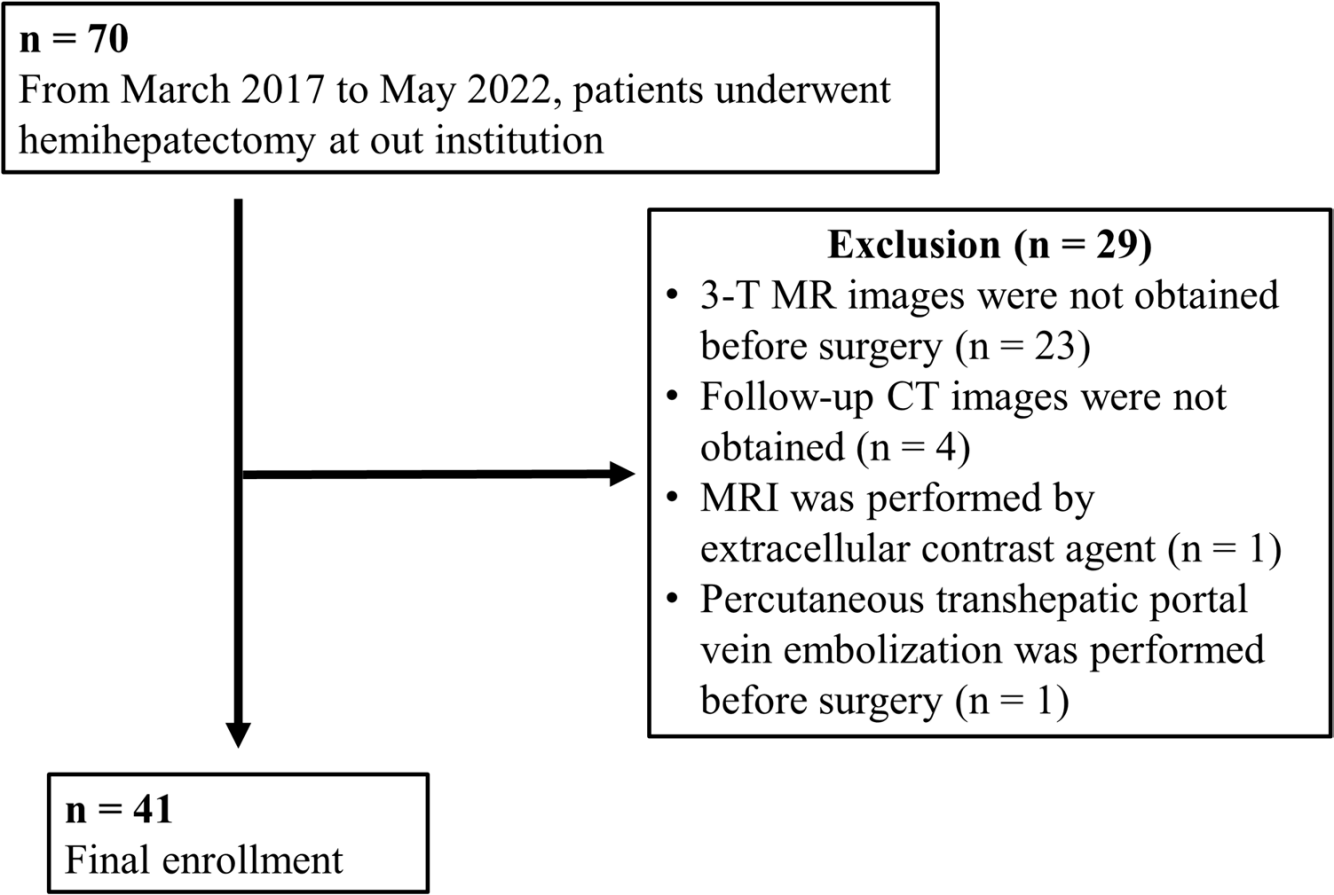
Flowchart of patient selection

The study design was approved by our institutional review board (No. 22057), and the requirement for informed consent was waived because of its retrospective design. Between March 2017 and May 2022, 70 consecutive patients underwent hemihepatectomy at our institution. The exclusion criteria were as follows: (1) 3 T MR images not obtained before surgery (n = 23); (2) follow-up CT images not obtained (n = 4); (3) MRI performed using an extracellular contrast agent (n = 1); and (4) percutaneous transhepatic portal vein embolization performed before MRI (n = 1). Figure 1 shows the flow-chart of the patient selection process. After these exclusions, 41 patients were enrolled retrospectively. Table 1 summarizes the patient characteristics.

**Table 1:** Characteristics of the 41 patients

Sex (male/female)	31/10
Age (y)*	70.8 ± 7.4
Etiology of liver disease	
Normal	27
Hepatitis C virus	8
Hepatitis B virus	4
Alcoholism	1
NASH	1
Tumor	
HCC	24
Cholangiocarcinoma	9
Metastasis	5
Inflammatory pseudotumor	3

*NASH* non-alcoholic steatohepatitis, *HCC* hepatocellular carcinoma, *ICC* intrahepatic cholangiocarcinoma

*Data are presented as the mean ± standard deviation

### CT protocols

Because we used a retrospective design, various types of CT scanners were used, and different protocols were followed for preoperative and postoperative CT. Preoperative dynamic CT studies were performed using a 64-slice (Aquilion; Canon Medical Systems, Otawara, Japan; n = 5), 80-slice (Aquilion Precision; Canon Medical Systems; n = 2), 256-slice (iQon Spectral CT; Philips Healthcare, Best, Netherlands; n = 16), or 320-slice (Aquilion One; Canon Medical Systems; n = 18) multidetector CT (MDCT) scanner. Scanning was performed before and after the administration of 600 mg I/kg of iodinated contrast material (Iopamiron 370 mg I/mL; Bayer, Osaka, Japan or Optiray 350 mg I/mL; Guerbet, Villepinte, France). The interval between preoperative CT and surgery was 32.2 ± 30.7 days (mean ± standard deviation [SD]). The contrast material was administered intravenously for 30 s. Two continuous arterial phases were scanned during a single breath-hold using a bolus-triggered technique (monitoring frequency from 10 s after contrast injection: 1 s; trigger threshold: an increase of 100 HU in the descending aorta; delay from trigger to initiation of scan: 15 s). The early arterial phase was used for CT angiography to plan the surgical procedure. Portal venous and delayed phases were acquired at 60 and 240 s, respectively. The scan parameters were a tube voltage of 120 kVp, automatically set mAs values, and a slice thickness of 1 mm.

Postoperative dynamic CT studies were performed at the first outpatient visit after surgery with a 64-slice (Brilliance 64: Philips, n = 5; Aquilion: Canon Medical Systems, n = 2), 80-slice (Aquilion Precision: Canon Medical Systems, n = 6), 256-slice (Brilliance iCT: Philips, n = 2; iQon Spectral CT: Philips, n = 10), or 320-slice (Aquilion One: Canon Medical Systems, n = 16) MDCT scanner. The interval between postoperative CT and surgery was 131.0 ± 72.7 days (mean ± SD). After precontrast images of the liver were obtained, each patient received 100 ml of intravenous non-ionic contrast material (Iopamiron 370 mg I/mL; Bayer, Osaka, Japan or Optiray 350 mg I/mL; Guerbet, Villepinte, France) at a rate of 3.0 ml/s using an automated power injector. An arterial phase image was obtained at a fixed delay of 45 s after the start of the contrast agent injection. The portal venous and delayed phases were acquired 70 and 240 s after the initiation of contrast agent injection, respectively. The scan parameters were as follows: a tube voltage of 120 kVp, automatically set mAs values, and a slice thickness of 5 mm.

### CT image analysis

Image analysis was performed by a board-certified surgeon (S.I. with 23 years of experience in liver surgery) and a board-certified radiologist (N.F. with 19 years of experience in abdominal imaging) who were blinded to the clinical and pathologic results in a consensus fashion. The portal phase data were transferred to a workstation and analyzed using a dedicated application (SYNAPSE VINCENT software, Fujifilm Medical Co. Ltd., Tokyo, Japan).

First, the entire liver and blood vessels (hepatic and portal veins) were automatically segmented. If liver benign lesions such as hepatic cysts or cavernous hemangiomas were present, the lesions were segmented semi-automatically and subtracted from the liver volume. In this study, we subtracted the volume of liver lesions with a diameter > 5 cm [15]. Second, using preoperative CT images, we calculated the total liver volume (LV), resected LV, and remnant LV in patients who underwent hemihepatectomy.

On postoperative CTs, the remnant LV was measured. Surgical records were retrieved, and corrections were made based on the surgical procedure, if necessary. We corrected the data on the application to achieve a precise final result.

We then determined the resection rate (RR) and liver regeneration index (LRI) using the following formulas: RR = Resected LV/Total LV × 100; LRI = (postoperative remnant LV−preoperative remnant LV)/preoperative remnant LV × 100 [16].

We also measured the spleen volume in the portal phase using the same workstation and application (SYNAPSE VINCENT software). Portal vein diameter was measured using the axial images of the portal phase.

### MRI protocols

Patients underwent gadoxetic acid-enhanced MRI at 3 T (Ingenia, Philips Healthcare) with a 32-channel torso-cardiac phased-array coil. The interval between preoperative MRI and surgery was 33.0 ± 35.8 days (mean ± SD).

The gadoxetic acid-enhanced MRI comprised a multiphase dynamic study, in which the arterial-dominant, portal, late, and hepatobiliary phases were imaged. Fat-suppressed gradient-echo T1-weighted images with enhanced three-dimensional T1 high-resolution isotropic volume excitation (eTHRIVE) were obtained for each phase. The total amount of gadoxetic acid (EOB･Primovist, Bayer, Osaka, Japan) based on the.patient’s body weight (0.025 mmol/kg) was intravenously injected for 5 s, followed by a 20 ml physiological saline infusion with an automatic injector (Nemoto Kyorindo, Tokyo). Scanning of the hepatobiliary phase was performed 20 min after the injection of the contrast agent. The imaging parameters of eTHRIVE were the following: repetition time (TR)/time to echo (TE)/flip angle (FA) = 3 ms/1.4 ms/10°, matrix: 252 × 200, field of view (FOV): 37.5 × 29.8 cm, SENSE factor: 1.8, slice thickness = 3 mm, gap =   −1.5 mm, linear k-space ordering, spectral attenuation with inversion recovery, acquired 133 sections, scan time: 17.9 s, and breath-holding.

Fat fraction and R2* map were added to our routine liver MRI protocol in 20 of 41 (48.8%) patients using the vendor-supplied modified Dixon quantification (mDIXON Quant) method. The imaging parameters of fat fraction and R2* were as follows: TR/TE/FA = 5.6 ms/0.97 ms/3°; scan mode: 3D; technique: fast field echo (FFE); matrix: 152 × 133; FOV: 33 × 33.2 cm; SENSE factor: 2; number of excitations (NEX): 1; slice thickness: 6 mm; gap: − 3 mm; scan time: 14.8 s; and breath-holding.

### MRI analysis

Preoperative MR image analysis was performed by two radiologists (N.F. and M.I. with 19 and 9 years of experience in abdominal imaging, respectively) who were blinded to the clinical and pathological results independently. The average values of the two readers were used.

**Fig. 2:**
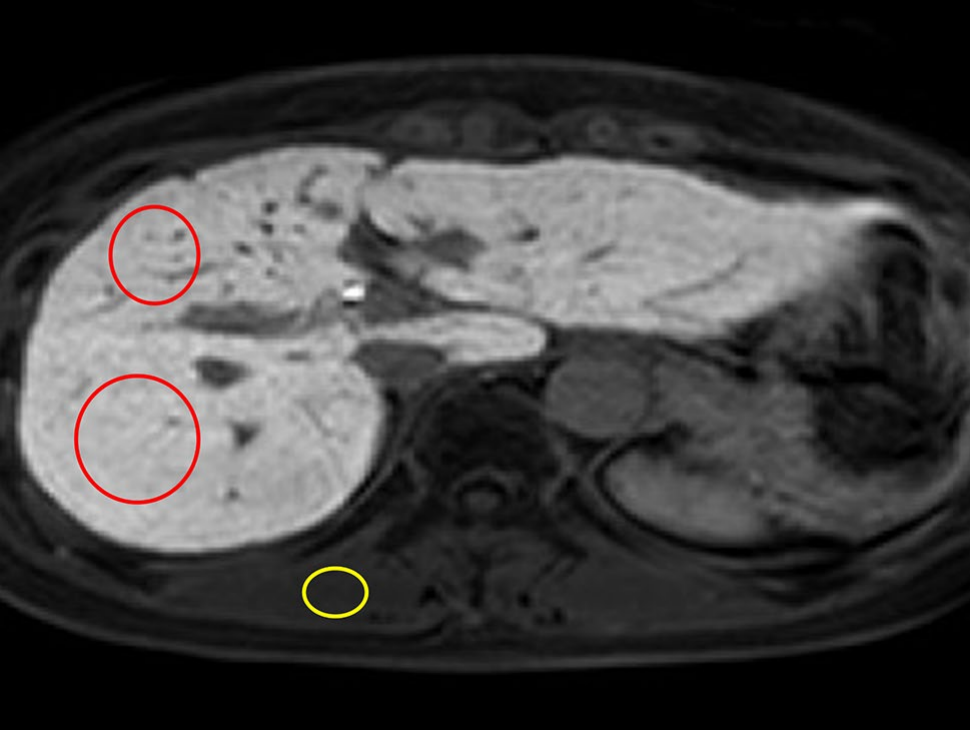
The hepatobiliary phase of gadoxetic acid-enhanced MRI. The signal intensities were measured by placing two round or oval regions of interest (ROIs) (red circles) on the remnant liver parenchyma, and one round or oval ROI on the erector spinae muscle (yellow circle) as large as possible

We used images in the precontrast and hepatobiliary phase for the evaluation of liver-to-erector spinae muscle signal intensity (SI) ratio (LMR) of the remnant liver. SIs were measured by placing two round or oval regions of interest (ROIs) on the remnant liver parenchyma and one ROI on the erector spinae muscle as large as possible while avoiding vessels (Fig. 2); for this, the commercially available PACS workstation (SYNAPSE, FujiFilm Medical) was used. Based on the average values of the two SIs of the remnant liver parenchyma and the erector spinae muscle, the LMR was calculated using the following equation:

$${\text{LMR}}\, = \,{\text{SI of the preoperative remnant liver}}/{\text{SI of the erector spinae muscle}}.$$

Finally, the ΔLMR was calculated using the following equation:

$$\Delta {\text{LMR}}\, = \,\left( {\text{LMR in the hepatobiliary phase - LMR in the precontrast image}} \right)/{\text{LMR in the precontrast image}}.$$

**Fig. 3:**
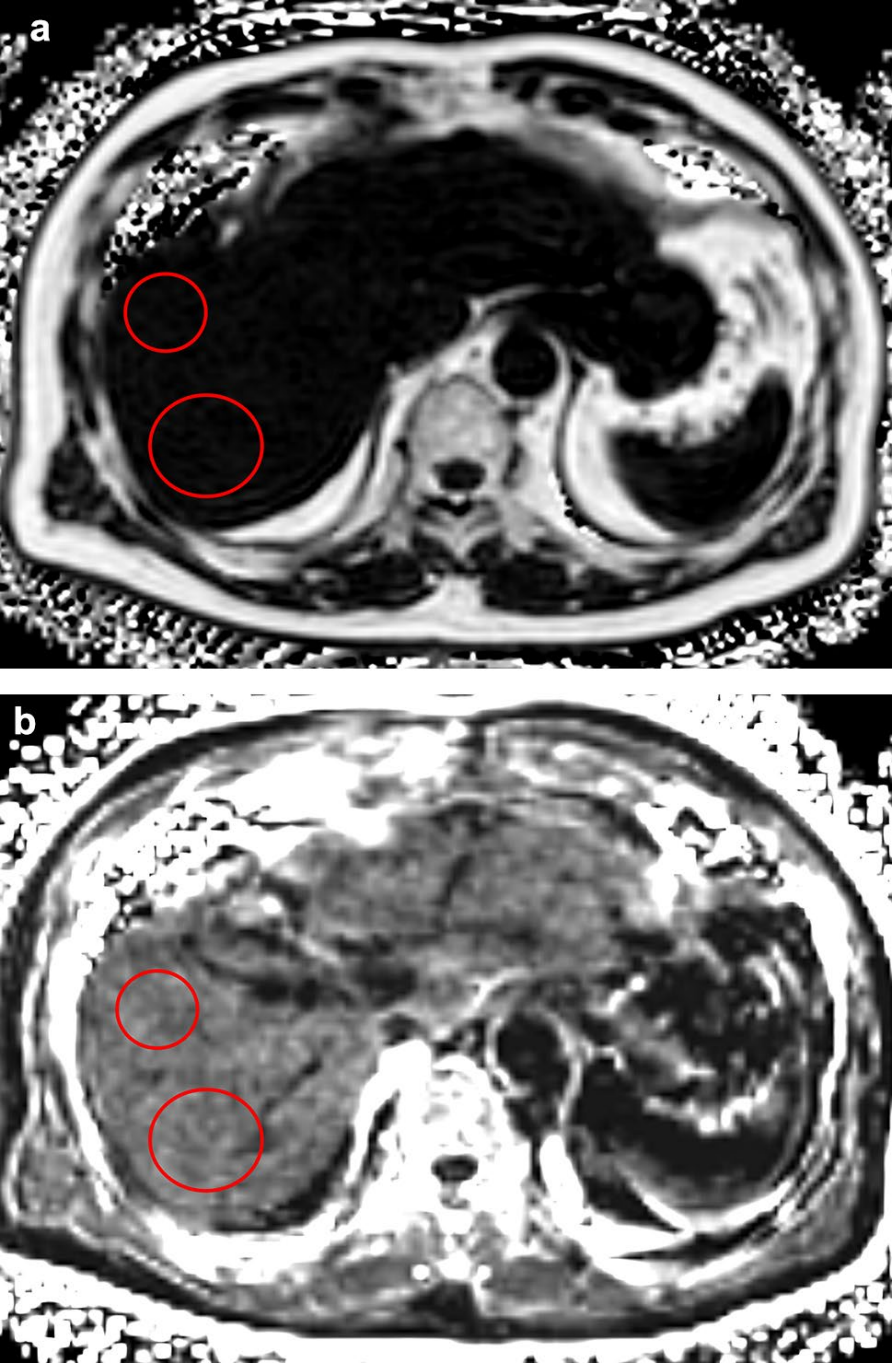
The fat fraction (**a**) and R2* (**b**) maps. The signal intensities were measured by placing two largest possible ROIs on the remnant liver parenchyma (red circles) while avoiding vessels and artifacts

For the fat fraction and R2* maps, radiologists placed two round or oval ROIs on the remnant liver parenchyma as large as possible avoiding vessels and artifacts, and the average values of the two SIs were used for the analysis (Fig. 3).

### Patient clinicopathological data

All 71 patients were clinicopathologically evaluated for liver dysfunction. Child–Pugh classification was defined as A, B, or C based on the definition [17]. The albumin-bilirubin (ALBI) score was in a nomogram ([log10 (17.1 × bilirubin (mg/dL) × 0.66)] + [10 × albumin (g/dL) ×  −0.085]), such that grade 1 is ≤  −2.60, grade 2 is <  −2.60 to ≤  −1.39, and grade 3 is >  −1.39 [18]. ALBI linear predictor (ALBI-LP) was calculated from the following equation: ALBI-LP = [0.66 × log10 bilirubin (μmol/L)] + [0.085 × albumin (g/L)] [19]. The fibrosis index based on four factors (FIB-4) was calculated using the following formula: [age (yr) x AST (IU/L)]/[PLT (10^9^/L) × (ALT (IU/L) (1/2)] [20]. ALBI-LP / total LV (L) and FIB-4 index/total LV were also evaluated. Indocyanine green (ICG) clearance tests were performed using 0.5 mg/kg per body weight Diagnogreen (Daiichi Pharmaceutical Cop., Tokyo, Japan). Blood samples were collected before and 15 min after ICG administration, and the retention rates 15 min after the injection (ICG-R15) were measured [21]. Additionally, we evaluated ICG-R15 / total LV (L), square root of ICG-R15 (√ICG-R15) and √ICG-R15/total LV (L). The pathological grade of necroinflammatory activity was scored using resected non-tumorous liver specimens according to the New Inuyama Classification: A0 (no necroinflammatory reaction), A1 (mild), A2 (moderate), or A3 (severe) [22]. The degree of liver fibrosis was scored as F0 (no fibrosis), F1 (fibrous portal expansion), F2 (bridging fibrosis), F3 (bridging fibrosis with architectural distortion), or F4 (liver cirrhosis) [22].

### Statistical analysis

The Kruskal–Wallis test or Mann–Whitney U test was used to analyze the correlation between categorical variables (Child–Pugh score and ALBI grade) and LRI. Pearson correlation coefficient analysis was used to analyze the correlation between continuous variables (the interval between postoperative CT and surgery, RR, preoperative portal vein diameter, spleen volume, ΔLMR, fat fraction, R2*, ALBI-LP, ALBI-LP/total LV, FIB-4 index, FIB-4 index / total LV, ICG-R15, ICG-R15 / total LV, √ICG-R15, and √ICG-R15 / total LV) and LRI. Spearman’s rank correlation test was used to analyze the correlation between the pathological stage and LRI. Significant values in the univariate analysis were entered into stepwise multiple regression analysis. For ΔLMR, fat fraction, and R2*, we calculated the intraclass correlation coefficient (ICC) with a 95% confidence interval (CI) to evaluate the inter-reader agreement. A correlation was considered excellent if the absolute value of the ICC was > 0.80, good if it was ≤ 0.80 to > 0.6, moderate if it was ≤ 0.6 to > 0.4, fair if it was ≤ 0.4 to > 0.2, and poor if it was ≤ 0.20. JMP 13.2.1 software (SAS Institute, Cary, NC, USA) was used for the analysis. Statistical significance was set at P < 0.05.

## Results

The clinicopathological liver function of 41 patients is summarized in Table 2.

**Table 2:** Clinicopathological liver function of the 41 patients

Child–Pugh score (A/B)	38/3
ALBI grade (1/2/3)	23/17/1
ALBI-LP*	−2.6 ± 0.4
ALBI-LP/total LV*	−2.2 ± 0.7
FIB-4 index*	2.8 ± 2.6
FIB-4 index/total LV*	2.3 ± 1.7
ICG-R15, %*	9.8 ± 5.5
ICG-R15/total LV*	8.3 ± 5.0
√ICG-R15, %*	3.0 ± 1.0
√ICG-R15/total LV*	2.5 ± 1.0
Background liver	
Inflammation	
A0	13
A1	21
A2	7
A3	0
Fibrosis	
F0	21
F1	7
F2	6
F3	4
F4	3

*ALBI* albumin-bilirubin, *ALBI-LP* albumin-bilirubin linear predictor, *LV* liver volume, *FIB-4* fibrosis index based on four factors, *ICG-R15* indocyanine green retention rate at 15 min

^*^Data are presented as mean ± standard deviation

For the CT parameters, LRI, RR, portal vein diameter, and spleen volume were 73.1 ± 35.5%, 42.4 ± 17.8%, 11.3 ± 2.0 mm, and 135.9 ± 64.5 mm^3^, respectively. For the MRI parameters, ΔLMR, fat fraction, and R2* were 0.79 ± 0.26, 5.6 ± 5.4%, and 73.1 ± 35.5 Hz, respectively. The ICC values of the ΔLMR, fat fraction, and R2* were 0.9574 (95% CI: 0.9215–0.9770), 0.9983 (95% CI: 0.9958- 0.9993), and 0.9948 (95% CI: 0.9870–0.9980), respectively, demonstrating excellent inter-reader agreement.

Univariate analysis revealed that RR and ΔLMR showed had a positive correlation with LRI (ρ = 0.7773, p < 0.001 and ρ = 0.4133, p = 0.0072, respectively). Spleen volume showed a negative correlation with LRI (ρ = -0.3138, p = 0.0486). Other clinicopathological parameters showed no significant correlation with LRI (Table 3). Stepwise multiple regression analysis showed RR and ΔLMR were independently correlated with LRI (β coefficient = 1.9653, p < 0.001 and β coefficient = 44.8771, p = 0.0198, respectively) (Table 4).

**Table 3:** Univariate analysis of correlations between liver regeneration index (LRI) and preoperative variables

Parameters	ρ	p-value
RR	0.7773	< 0.001*
Portal vein diameter	−0.0640	0.6911
Spleen volume	−0.3138	0.0486*
ΔLMR	0.4133	0.0072*
Fat fraction	−0.2252	0.3385
R2*	−0.1348	0.5709
Child–Pugh score	N/A	0.3415
ALBI grade	N/A	0.4826
ALBI-LP	0.0556	0.7300
ALBI-LP/total LV	0.0129	0.9361
FIB-4 index	−0.1633	0.3075
FIB-4 index/total LV	−0.1795	0.2615
ICG-R15, %	−0.2050	0.1986
ICG-R15/total LV	−0.1214	0.4497
√ICG-R15, %	−0.2333	0.1422
√ICG-R15/total LV	−0.1242	0.4390
A	0.0479	0.7693
F	0.0806	0.6211
Interval between postoperative	0.1824	0.2537
CT and surgery (days)		

*LRI* liver regeneration index, *RR* resection rate, *LMR* liver-to-muscle signal intensity ratio, *ALBI grade* albumin-bilirubin grade, *ALBI-LP* albumin-bilirubin linear predictor, *LV* liver volume, *FIB-4* fibrosis index based on four factors, *ICG-R15* indocyanine green retention rate at 15 min

*Statistically significant

**Table 4:** Multivariate analysis of correlations between LRI and preoperative variables

Parameters	β coefficient	p-value
RR	1.9635	< 0.001*
Spleen volume	N/A	0.4258
ΔLMR	44.8771	0.0198*

*LRI* liver regeneration index, *RR* resection rate, *LMR* liver-to-muscle signal intensity ratio

*Statistically significant

**Fig. 4:**
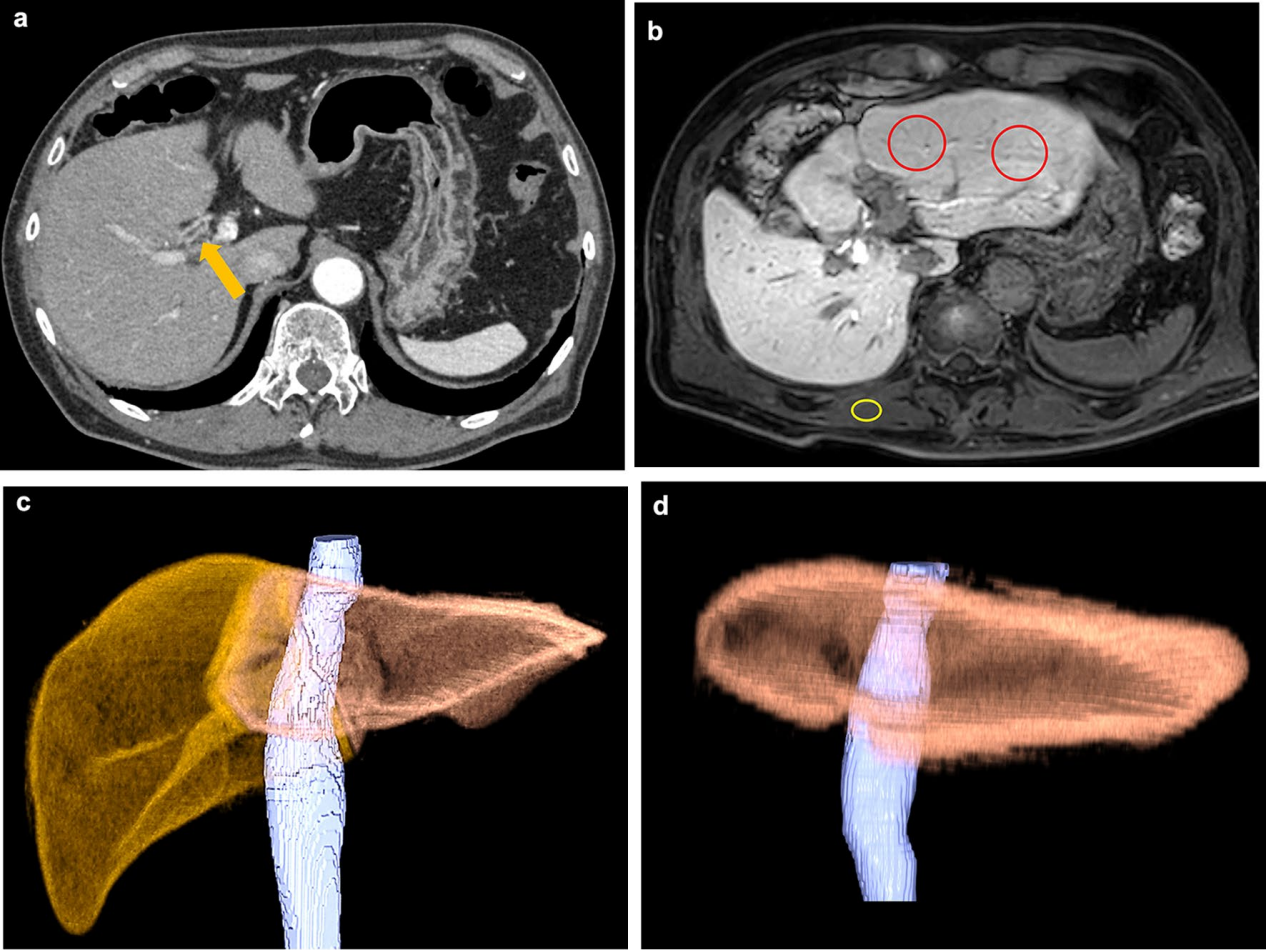
A 79-year-old man with right hilar cholangiocarcinoma. **a** Arterial-dominant phase of dynamic CT showing wall thickening of the right hepatic duct (arrow). **b** The hepatobiliary phase of gadoxetic acid-enhanced MRI. ΔLMR of the left lobe was 0.90. **c** Preoperative CT volumetry. The left and total liver volumes were 368 and 1021 mm^3^, respectively. The resection rate (RR) was 64.0%. **d** Postoperative CT volumetry was performed 197 days after the right hemihepatectomy. The left liver volume was 915 mm^3^ and the liver regeneration index (LRI) was 148.6%

**Fig. 5:**
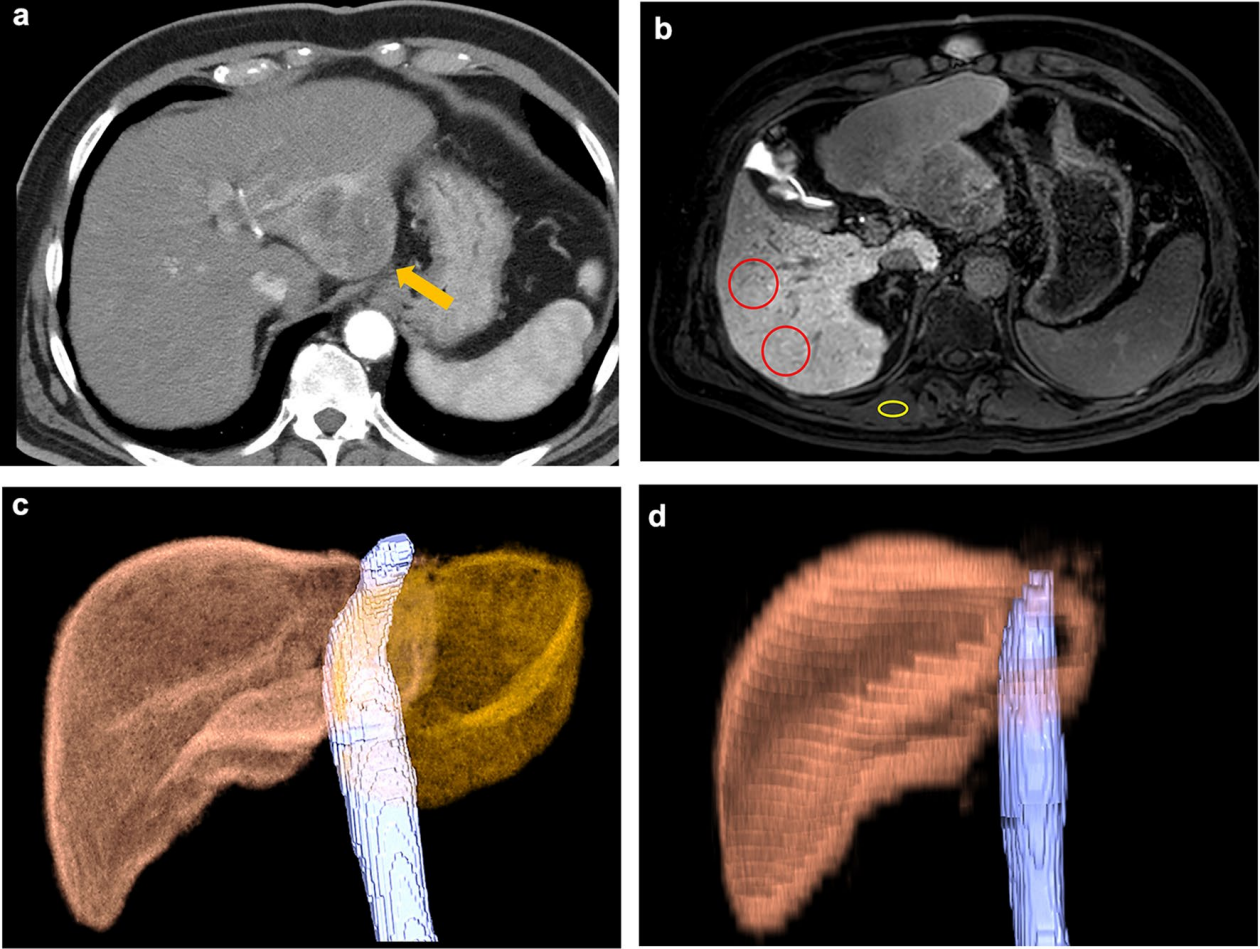
A 79-year-old man with hepatocellular carcinoma in the left lobe of the liver. **a** Arterial-dominant phase of dynamic CT shows heterogeneously enhanced mass in the left lateral segment of the liver (arrow). **b** The hepatobiliary phase of gadoxetic acid-enhanced MRI. ΔLMR of the right lobe was 0.33. **c** Preoperative CT volumetry. The right and total liver volumes were 620 and 944 mm^3^, respectively. The RR was 34.3%. **d** Postoperative CT volumetry performed 86 days after the left hemihepatectomy. The right liver volume was 913 mm^3^ and the LRI was 47.3%

Figures 4 and 5 present representative cases.

## Discussion

In the present study, two imaging parameters (RR and ΔLMR) were found to be closely associated with LR in patients undergoing hepatectomy. It has been well-reported that a larger liver resection promotes LR because of the greater concentration of cytokines produced due to the metabolic requirements, increased blood flow ratio, and larger space for regeneration [23, 24]. These previous results are consistent with those in our study, in which RR showed a positive correlation with LRI. Furthermore, in our study, we found that ΔLMR was another significant predictor of LR. In addition to detection or characterization of liver tumors, several studies have demonstrated the usefulness of gadoxetic acid-enhanced MRI for determining liver function using several parameters derived from pre- and post-contrast T1 sequences [13, 25]. Briefly, the uptake of gadoxetic acid in the liver parenchyma decreases as liver function worsens. Liver function has also been reported to cause poor LR [8, 9]. These results explain our finding that higher ΔLMR was significantly associated with a higher LRI. It has been reported that a lower uptake of gadoxetic acid correlates with increased liver-related morbidity after hepatic resection [26]. Considering these results, the evaluation of gadoxetic acid-enhanced MRI before surgery is clinically important and may serve as a preoperative predictor of patient prognosis after hepatic resection.

Another interesting finding in this study was that the clinical liver function (Child–Pugh score, ALBI grade, ALBI-LP, FIB-4 index, ICG-R15, and √ICG-R15) and the pathological grade of necroinflammatory activity and fibrosis were not significantly correlated with LRI. These results differ from those in previous reports [7–9]. We suspect that the reason for this difference is the relatively well-preserved liver function of patients who underwent hemihepatectomy. Especially, only 3 of 41 (7.3%) patients were diagnosed with liver cirrhosis. In other words, gadoxetic acid-enhanced MRI may be a useful imaging biomarker that can evaluate both liver function and pathology simultaneously and is a better predictor of LR than traditional clinicopathological factors in patients with preserved liver function. Additionally, it has been reported that the volume-integrated index of signal intensity of the hepatobiliary phase reflects the overall hepatic reserve expressed as ICG plasma disappearance rate (ICG-PDR) at a fixed dose of gadoxetic acid per body weight [27]. We also analyzed the correlation between clinical parameters corrected for liver volume (ALBI-LP/total LV, FIB-4 index/total LV, ICG-R15/total LV, and √ICG-R15/total LV) and LRI, but these parameters showed no significant correlation with LRI. Thus, uncorrected hepatic signal intensity (ΔLMR) can be considered to reflect regional hepatic blood flow and hepatocyte function rather than total liver function.

Previous studies have reported that MRI parameters such as MR elastography [28] and intravoxel incoherent motion (IVIM) [16] are also useful for the preoperative evaluation of LR. However, these methods may be expensive, cumbersome, inaccessible, or require additional hardware and/or software. In contrast, gadoxetic acid-based MRI is now widely used to detect and characterize lesions in patients with known or suspected hepatic mass. For this reason, we believe that our method may be practical for evaluating preoperative liver parenchyma and may be readily available to most clinicians.

Liver fat content is a predictor of worse LR after percutaneous Transhepatic Portal vein Embolization [29]. Iron overload has also been reported to cause liver fibrosis [30], which may inhibit LR. Therefore, we analyzed the fat fraction and R2* of the liver parenchyma using mDIXON Quant, which is useful for the accurate quantification of fat and iron deposition in the liver [31, 32]. However, fat fraction and R2* did not show a significant correlation with LRI in our study. We were able to measure fat fraction or R2* in only 20 of 41 (48.8%) patients, so further investigations with a larger number of patients are needed.

Our study had some limitations. First, our retrospective design and relatively small number of patients from a single institution may have led to a selection bias. Additionally, various CT scanners were used in this study. Second, the imaging protocols for preoperative and postoperative CT differed. In particular, the difference in slice thickness (1 mm vs. 5 mm) may have affected our results. Third, the interval between preoperative and postoperative imaging was not uniform. Although the interval between postoperative CT and surgery showed no significant correlation with LRI, a prospective study with uniform interval imaging, especially for postoperative CT, is needed for a more accurate evaluation of LR. Notably, Nadalin et al. reported that volumetric restoration of the liver parenchyma occurs within 2–3 weeks after major hepatectomy [33]. In the present study, the interval between postoperative CT and surgery was 131.0 ± 72.7 days (mean ± SD), which could be a tolerable range for LR. Fourth, the placement of ROIs was subjective. Additionally, the evaluation could have been affected by small vessels and bile ducts. To overcome these issues, two experienced abdominal radiologists performed the image analysis independently and excellent interobserver agreement was obtained when we evaluated ΔLMR, fat fraction, and R2*. Fifth, we could not evaluate ICG-PDR, because blood samples were only collected at 15-min intervals following ICG administration.

In conclusion, ΔLMR and RR showed a positive correlation with LRI in patients undergoing hemihepatectomy. ΔLMR measured from gadoxetic acid-enhanced MRI may serve as a preoperative predictor of LR.
